# 

**Published:** 1899-06-10

**Authors:** 


					THE HOSPITAL. " The standard of highest purity." Lancet. June 10th, 1899.
^CADBURY'S H B* CADBURY'S 0
COCOA % ^ COCOA
ABSOLUTELY PURE* NO DRUGS OR ALKALIES USED.
? _  DRIYICH HOSFITAU
rCLAsban Western Infirmary
No. 603 Vol. XXVI. |JSS?*?I Saturday,
June 10th, 1899.
PRICE TWOPENCE.

				

